# Diagnostic value of D-dimer in screening for deep vein thrombosis after total joint arthroplasty: a meta-analysis

**DOI:** 10.3389/fsurg.2026.1783183

**Published:** 2026-07-14

**Authors:** Xinzhen Ding, Hubing Wu, Qionglin Huang

**Affiliations:** 1 Department of Laboratory, Yueqing People’s Hospital, Yueqing, Zhejiang, China; 2 Department of Orthopedics, Yueqing Third People’s Hospital, Yueqing, Zhejiang, China

**Keywords:** D-dimer, meta-analysis, sensitivity and specificity, total joint arthroplasty, venous thrombosis

## Abstract

**Background:**

Deep vein thrombosis (DVT) is a common and serious complication following total hip arthroplasty (THA) and total knee arthroplasty (TKA). Although plasma D-dimer is widely used to exclude thrombotic events, its diagnostic accuracy in postoperative populations remains controversial due to nonspecific elevation caused by surgical trauma. This meta-analysis aimed to systematically evaluate the diagnostic performance of D-dimer for screening DVT after total joint arthroplasty (TJA).

**Methods:**

PubMed, Web of Science, Wiley Library, China National Knowledge Infrastructure (CNKI), Wanfang Data, and VIP databases were searched from inception to November 2025 for studies that investigated the diagnostic accuracy of D-dimer for detecting DVT after TJA. Study quality was assessed using the QUADAS-2 tool. A bivariate mixed-effects model was applied to pool sensitivity, specificity, positive likelihood ratio (PLR), negative likelihood ratio (NLR), and diagnostic odds ratio (DOR). Summary receiver operating characteristic (SROC) curves were constructed and the area under the curve (AUC) was calculated using Stata version 14.0.

**Results:**

A total of 11 studies involving 7,123 patients were included. The pooled sensitivity and specificity of D-dimer for diagnosing postoperative DVT after TJA were 0.76 (95% CI: 0.64–0.85) and 0.86 (95% CI: 0.55–0.97), respectively. The pooled PLR was 5.24 (95% CI: 1.30–21.08), the NLR was 0.28 (95% CI: 0.16–0.49), and the DOR was 18.59 (95% CI: 2.94–117.42). The AUC of the SROC curve was 0.83 (95% CI: 0.80–0.86). Substantial heterogeneity was observed among studies (I^2^ > 98%), which was mainly attributable to differences in diagnostic cutoff values.

**Conclusions:**

D-dimer demonstrates moderate diagnostic potential for screening DVT after TJA, with its primary value lying in ruling out DVT when results are negative. However, the current pooled estimates do not support a uniform diagnostic threshold or the use of D-dimer as a standalone diagnostic tool. Given the substantial heterogeneity across studies and the variability in specificity due to postoperative physiological elevation, the test should only be considered as part of a risk-stratified screening strategy, ideally in combination with clinical risk assessment models or using procedure- and context-specific cutoff values. Further prospective studies are needed to validate such integrated approaches before clinical implementation.

## Introduction

With the intensification of population aging, total hip arthroplasty (THA) and total knee arthroplasty (TKA) have become the most effective means for treating end-stage joint diseases (1, 2). However, deep vein thrombosis (DVT) remains one of the most common and serious complications following joint replacement surgery (3). Despite the widespread application of perioperative anticoagulation prophylaxis, the incidence of DVT after total joint arthroplasty (TJA) remains high (4). If not diagnosed and treated promptly, DVT may dislodge and lead to fatal pulmonary embolism (PE), posing a serious threat to patients’ lives (5, 6). Therefore, early, rapid, and accurate screening for DVT is crucial for improving patient outcomes. Although venography is regarded as the “gold standard” for diagnosing DVT, its invasive nature, nephrotoxicity from contrast agents, and high cost make it difficult to implement routinely in clinical practice. Doppler ultrasound, as the preferred non-invasive imaging modality, exhibits high specificity but is operator-dependent, and its use for screening asymptomatic patients is associated with high costs and long durations (7). Consequently, there is an urgent clinical need for a simple, economical, and non-invasive biomarker for early screening and risk stratification of postoperative DVT, in order to reduce unnecessary imaging examinations.

Plasma D-dimer, a specific degradation product of cross-linked fibrin, reflects the hypercoagulable state and secondary hyperfibrinolysis in the body and has been widely used to exclude venous thromboembolism (VTE) in outpatient and emergency settings (8). However, the diagnostic value of D-dimer in major orthopedic surgery has been highly controversial. The inflammatory response and coagulation system activation triggered by surgical trauma lead to a physiological, significant increase in postoperative D-dimer levels. This “non-thrombotic elevation” substantially reduces its diagnostic specificity, resulting in a large number of false positives (9, 10). Whether conventional diagnostic thresholds (e.g., 0.5 mg/L) remain applicable in the postoperative population, and whether age-adjusted or higher cut-offs are needed to balance sensitivity and specificity, remains undetermined (4, 9–13). Although a limited number of studies have explored the application of D-dimer after joint arthroplasty, significant variations exist among studies in terms of sample size, timing of testing, and cut-off values, leading to inconsistent conclusions. In light of this, this study aims to comprehensively evaluate the overall diagnostic accuracy of D-dimer for screening DVT after total joint arthroplasty through a systematic review and meta-analysis. Furthermore, it will explore the impact of different surgical types and cut-off values on diagnostic performance, aiming to provide evidence-based medical support for developing rational clinical screening strategies.

## Methods

### Literature search strategy

The literature search was conducted across multiple renowned domestic and international databases, including PubMed, Web of Science, Wiley Library, China National Knowledge Infrastructure (CNKI), WanFang, and VIP, covering the period from inception to November 2025. The primary English search terms included: “Deep Vein Thrombosis”, “Venous Thromboembolism”, “Arthroplasty”, “Replacement”, “Total Hip Arthroplasty”, “Total Knee Arthroplasty”, as well as “D-dimer”, “Fibrin fragment”, “Sensitivity”, “Specificity” and “Accuracy”. Additionally, the reference lists of included studies will be manually reviewed to supplement potentially relevant research and prevent omissions. The entire screening process will strictly adhere to the PRISMA (Preferred Reporting Items for Systematic Reviews and Meta-Analyses) guidelines (14) to ensure transparency and consistency in literature screening and analysis. The complete search strategies for all databases are provided in Supplementary Material 1.

### Inclusion and exclusion criteria

Strict screening criteria were formulated based on the PICOS principle (Participants, Index test, Comparator, Outcomes, Study design). Inclusion criteria: (1) Participants: Patients undergoing total joint arthroplasty (TJA), including total hip arthroplasty (THA), total knee arthroplasty (TKA), and femoral head replacement (FHR); (2) Index test: Quantitative plasma D-dimer testing (preoperative or postoperative) with a clearly defined diagnostic cut-off value; (3) Reference standard: Venography or Doppler ultrasonography served as the gold standard for DVT diagnosis; (4) Outcome measures: The study provided complete data on true positives (TP), false positives (FP), false negatives (FN), and true negatives (TN), or sufficient data to construct a 2 × 2 contingency table. Exclusion criteria: (1) Reviews, conference abstracts, case reports, editorials, and animal studies; (2) Studies with excessively small sample sizes (<10 cases); (3) Literature from which complete 2 × 2 table data could not be extracted or with duplicate publication of data.

### Data extraction and quality assessment

Two researchers independently performed literature screening and data extraction based on a pre-designed standardized form. Extracted content included: first author, publication year, country, study design type (prospective/retrospective), sample size, surgical method, cut-off value, and diagnostic outcome data, including sensitivity, specificity, and the area under the ROC curve (AUC). Discrepancies were resolved through discussion or arbitration by a third senior researcher. The methodological quality of included studies was assessed using the Quality Assessment of Diagnostic Accuracy Studies-2 (QUADAS-2) tool, evaluating the risk of bias and applicability concerns across four domains: patient selection, index test, reference standard, and flow and timing.

### Statistical analysis

All statistical analyses were performed using the Midas module within Stata 14.0 software (Stata Corp, USA). Considering the frequent negative correlation (threshold effect) between sensitivity and specificity in diagnostic meta-analyses, a bivariate mixed-effects regression model was employed for data synthesis. The primary statistical metrics included: pooled sensitivity, pooled specificity, negative likelihood ratio (NLR), and diagnostic odds ratio (DOR), along with their corresponding 95% confidence intervals (CI). A summary receiver operating characteristic curve (SROC) was plotted, and the AUC was calculated to comprehensively evaluate the overall diagnostic performance of D-dimer: an AUC of 0.5–0.7 indicates low diagnostic value, 0.7–0.9 indicates moderate value, and >0.9 indicates high value. Heterogeneity was assessed by visual inspection of forest plots and calculation of the I^2^ statistic. An I^2^ > 50% or *P* < 0.05 indicated significant heterogeneity. Furthermore, a Fagan's nomogram was plotted to evaluate the clinical value of D-dimer testing in altering the post-test probability of DVT. Potential publication bias was assessed using Deeks’ funnel plot asymmetry test, with *P* < 0.10 indicating significant publication bias.

Potential sources of clinical heterogeneity, including differences in the timing of D-dimer measurement (preoperative vs. postoperative), diagnostic cut-off values, and surgical types, were acknowledged. However, due to the limited number of studies in relevant subgroups (e.g., only four studies measured D-dimer preoperatively), formal subgroup analyses or meta-regression were not performed to avoid unstable estimates and loss of statistical power. Instead, these sources of heterogeneity are thoroughly addressed in the Discussion as key limitations and contextual considerations for interpreting the pooled results.

In diagnostic meta-analysis, the bivariate mixed-effects model separately estimates sensitivity (based on DVT-positive subjects) and specificity (based on DVT-negative subjects). Studies with zero DVT events contribute no data to the DVT-positive group; therefore, they do not influence the pooled sensitivity estimate but continue to contribute to the specificity estimate through true-negative cases. Such studies were retained in the primary analysis because excluding them would unnecessarily reduce sample size and could introduce bias without improving the accuracy of the sensitivity estimate.

## Results

### Literature screening and basic characteristics of included studies

**Figure 1:**
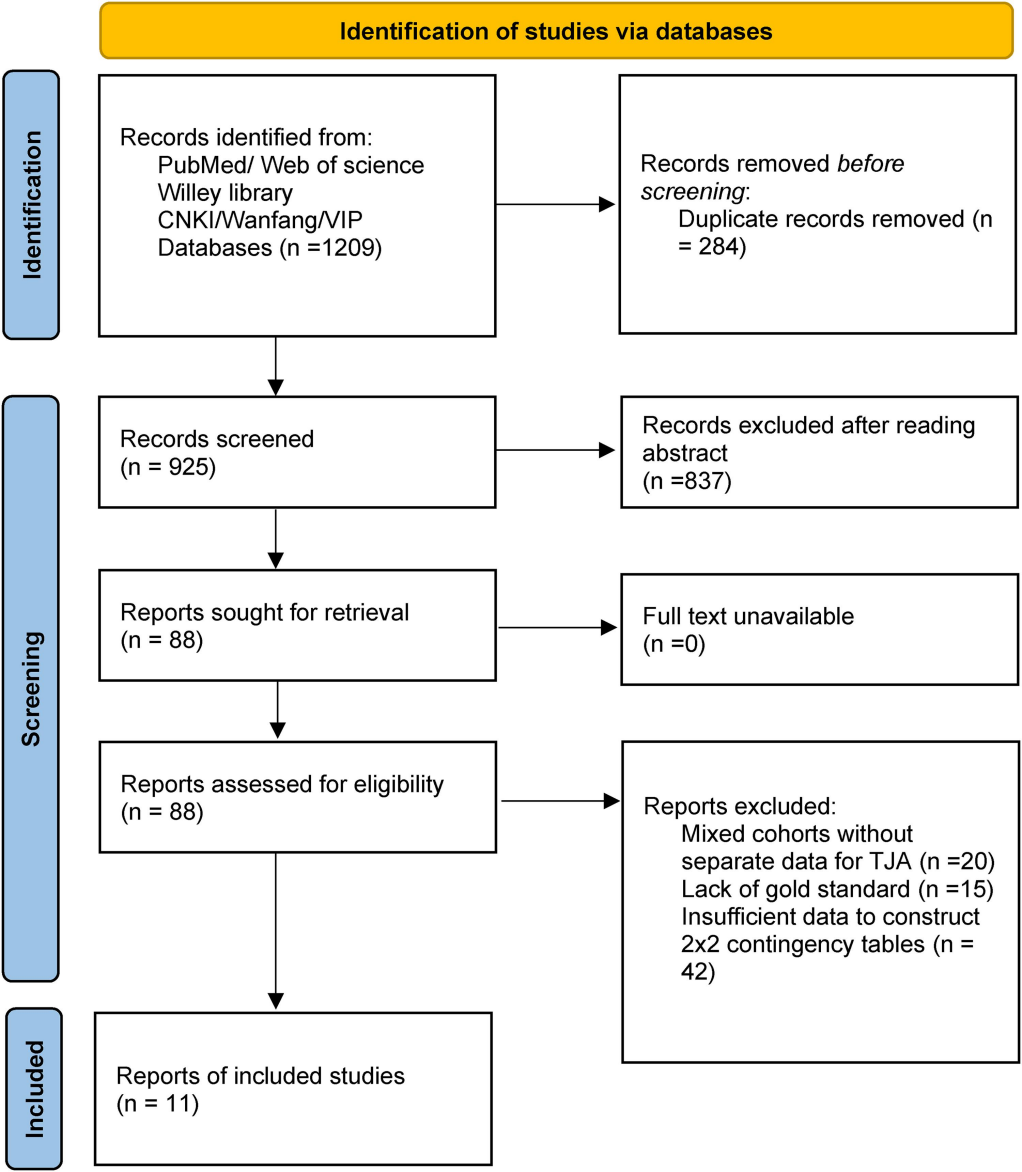
Flowchart of literature screening.

This meta-analysis ultimately included 11 studies (4, 9–12, 15–20) meeting the criteria (Figure 1), covering various types of joint replacement surgeries, including THA, TKA, and femoral head replacement (FHR). The involved regions included China, Thailand, Japan, and the UK. The majority of the studies were retrospective in design, with one study being prospective. The detailed characteristics are presented in Table 1. The methodological quality of the included studies, assessed using the QUADAS-2 tool, is summarized in Table 2. Overall, all studies had low risk of bias in the reference standard and flow/timing domains. However, a high risk of bias was observed in the patient selection domain for the majority of studies, primarily due to their retrospective design and the use of case-control or non-consecutive patient enrollment. The index test domain also showed some concerns, mainly related to the lack of prespecified cutoff thresholds. Among the included studies, one study by Rafee et al. (18) reported zero postoperative DVT events. As such, it contributed no data to the estimation of sensitivity but provided 70 true-negative results for the specificity estimate. This study was retained in the primary analysis according to the statistical rationale described in the Methods section.

**Table 1:** Basic characteristics of included studies.

Study	Year	Country	Study Design	Sample Size (DVT vs. non-DVT)	Surgical Procedure	DVT Incidence, %	Timing of D-dimer Measurement	Critical Cutoff Value	Reference Standard	AUC	Sensitivity	Specificity
Chen, C. (9)	2008	China	Retrospective	31/47	Total Knee Arthroplasty	68.0%	Postoperative day 7	0.2 mg/L	Venography	0.69	0.68	0.55
Chotanaphuthi, T. (10)	2009	Thailand	Retrospective	31/28	Total Knee Arthroplasty	52.0%	Not reported (excluded from timing subgroup)	0.50 mg/L	Venography	0.52	0.58	0.46
Huang, G. (11)	2024	China	Retrospective	62/82	Femoral Head Replacement	43.1%	Postoperative day 1–3	0.5 mg/L	Doppler Ultrasound	0.84	0.82	0.75
Imai, N. (12)	2017	Japan	Retrospective	13/211	Total Hip Arthroplasty	5.8%	Preoperative	0.185 mg/L	Doppler Ultrasound	0.72	0.60	0.82
Luo, H. (4)	2023	China	Retrospective	46/104	Total Hip Arthroplasty	30.6%	Postoperative day 3	0.595 mg/L	Doppler Ultrasound	0.97	0.89	1.00
Bai, L. (15)	2016	China	Retrospective	150/150	Hip Arthroplasty	50.0%	Not reported	-	Doppler Ultrasound	0.86	0.87	0.76
Xin, H. (16)	2018	China	Retrospective	19/41	Hip Arthroplasty	31.6%	Postoperative day 1, 3, 7	0.50 mg/L	Doppler Ultrasound	0.95	0.89	1.00
Qiao,L. (17)	2024	China	Retrospective	303/2570	Total Knee Arthroplasty	10.6%	Preoperative	0.5 mg/L	Doppler Ultrasound	0.56	0.58	0.51
Rafee, A. (18)	2008	UK	Prospective	0/70	Total Hip/Knee Arthroplasty	0.0%	Postoperative day 5–7	>0.59 mg/L	Venography	N/A	1.00	0.00
Wu, J. (19)	2021	China	Retrospective	39/367	Hip/Knee Arthroplasty	9.6%	Preoperative	0.845 mg/L	Doppler Ultrasound	0.69	0.41	0.66
Zhang, K. (20)	2021	China	Retrospective	280/2479	Joint Arthroplasty	34.4%	Preoperative	0.5 mg/L	Doppler Ultrasound	0.73	0.85	0.22

Preoperative indicates D-dimer measured before surgery; Postoperative indicates measurement after joint arthroplasty.

**Table 2:** Quality assessment of included literature.

Included Studies	Risk of Bias			Applicability Concerns			
	Case Selection	Index test	Reference Standard	Flow and Timing	Case Selection In	Index test	Gold standard
Bai, L. 2016 (15)	H	U	L	L	L	L	L
Xin, H. 2018 (16)	H	H	L	L	L	L	L
Chen, C. 2008 (9)	H	H	L	L	L	L	L
Chot. T. 2009 (10)	H	L	U	L	L	L	L
Huang, G. 2024 (11)	H	U	L	L	L	L	L
Imai, N. 2017 (12)	H	H	L	L	L	L	L
Luo, H. 2023 (4)	H	U	L	L	L	L	L
Qiao, L. 2024 (17)	H	L	L	L	L	L	L
Rafee, A. 2008 (18)	L	L	L	L	L	L	L
Wu, J. 2021 (19)	H	H	L	L	L	L	L
Zhang, K. 2021 (20)	H	H	L	L	L	L	L

L, (low risk); H, (high risk); U, (unclear risk).

### Overall diagnostic accuracy

**Figure 2:**
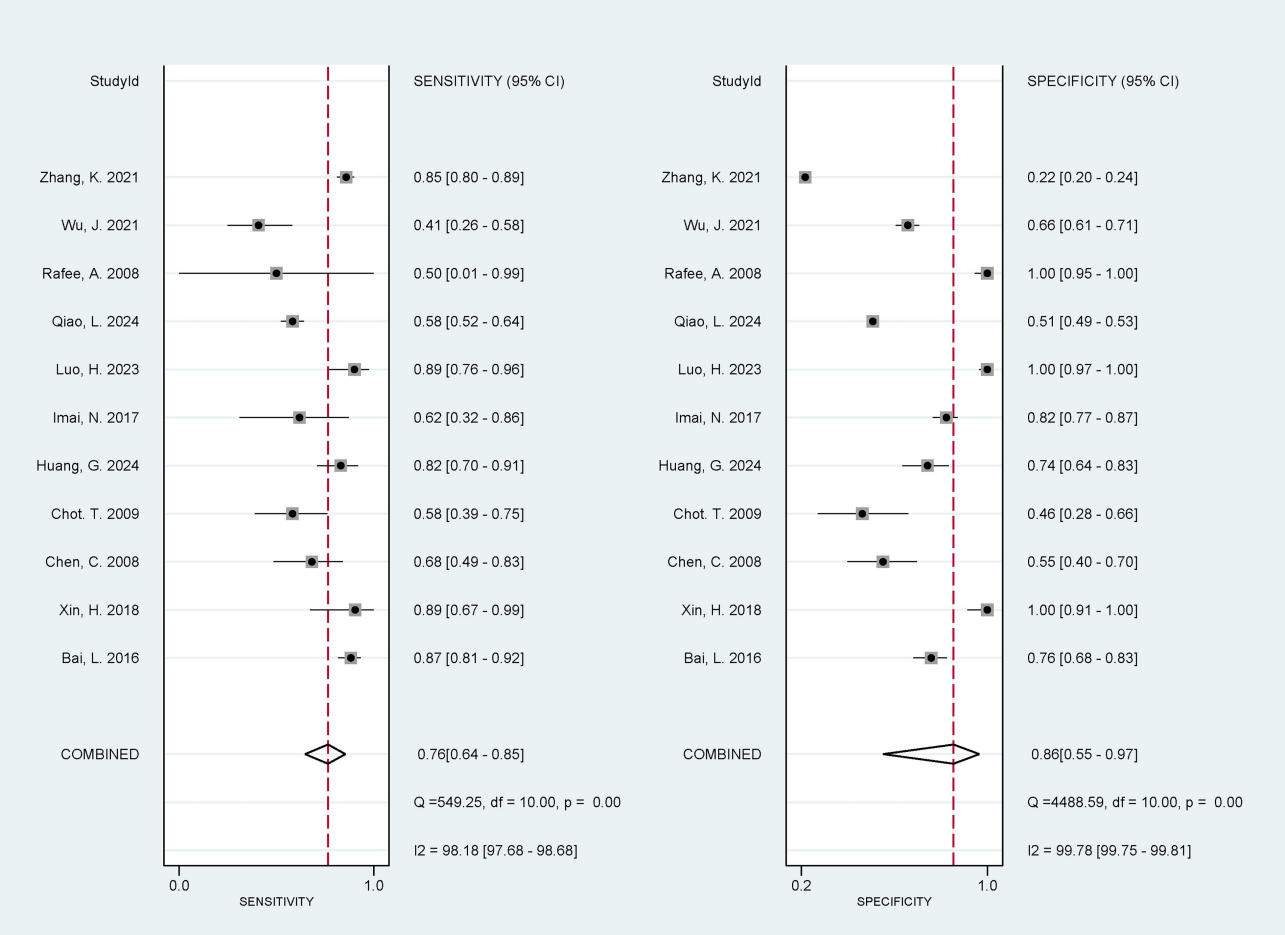
Forest plot of the sensitivity and specificity of D-dimer for diagnosing deep vein thrombosis after artificial joint replacement.

**Figure 3:**
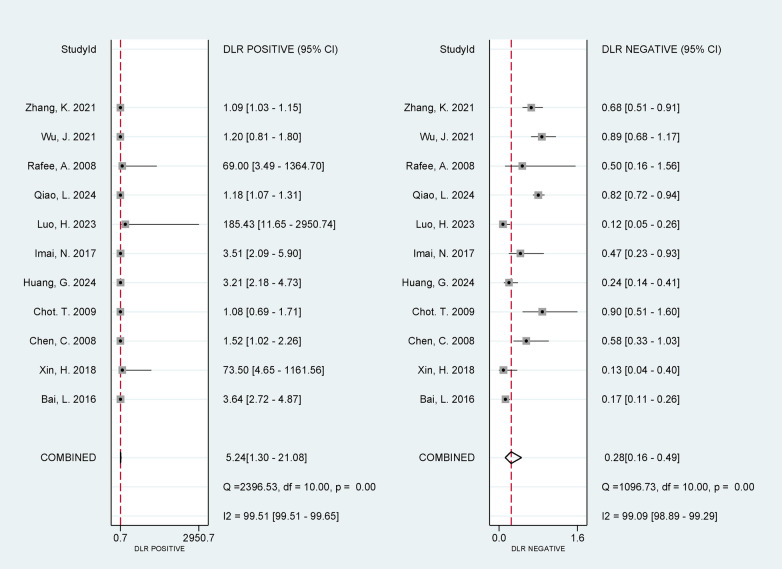
Forest plot of the PLR and NLR of D-dimer for diagnosing deep vein thrombosis after artificial joint replacement.

**Figure 4:**
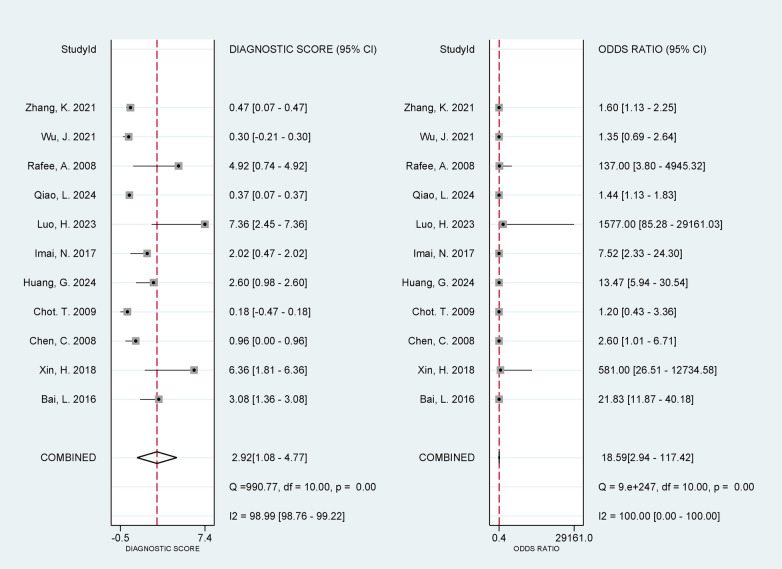
Forest plot of the DOR of D-dimer for diagnosing deep vein thrombosis after artificial joint replacement.

**Figure 5:**
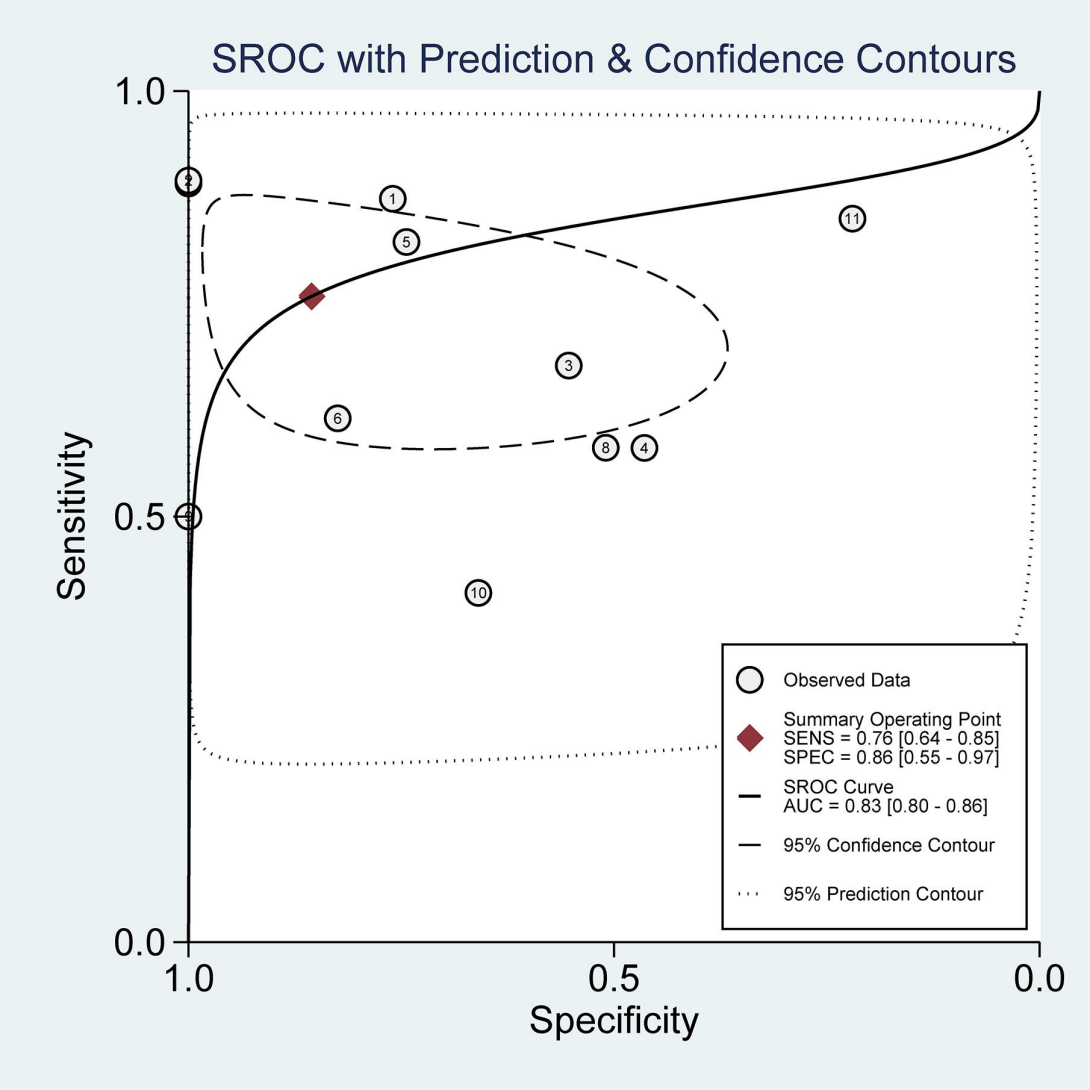
SROC curve of D-dimer for diagnosing deep vein thrombosis after artificial joint replacement.

The meta-analysis results demonstrated that the pooled sensitivity of D-dimer for screening DVT after TJA was 0.76 (95% CI: 0.64–0.85), and the pooled specificity was 0.86 (95% CI: 0.55–0.97). The PLR was 5.24 (95% CI: 1.30–21.08), and the NLR was 0.28 (95% CI: 0.16–0.49). The DOR was 18.59 (95% CI: 2.94–117.42). The SROC showed an AUC of 0.83 (95% CI: 0.80–0.86). The forest plot revealed significant statistical heterogeneity among the included studies. The I^2^ for sensitivity was 98.18% (95% CI: 97.68–98.68), and for specificity, it was 99.78% (95% CI: 99.75–99.81); the Q-test indicated *P* < 0.001 for both. Substantial heterogeneity was observed among studies (I^2^ > 98% for both sensitivity and specificity). This heterogeneity is likely attributable to multiple factors, including variations in diagnostic cut-off values, differences in surgical procedures (THA vs. TKA), and importantly, the timing of D-dimer measurement relative to surgery (preoperative in four studies vs. postoperative in seven studies). The SROC curve displayed an asymmetric “shoulder” shape, and the prediction contour was relatively wide, further confirming variability in precision across studies. The forest plots for PLR and NLR also showed very high heterogeneity (I^2^ > 99%), which may be related to differences in D-dimer cutoff values, detection time points, and surgical types among the studies (Figures 2–5), indicating that D-dimer has good overall diagnostic performance after joint arthroplasty.

### Clinical utility

**Figure 6:**
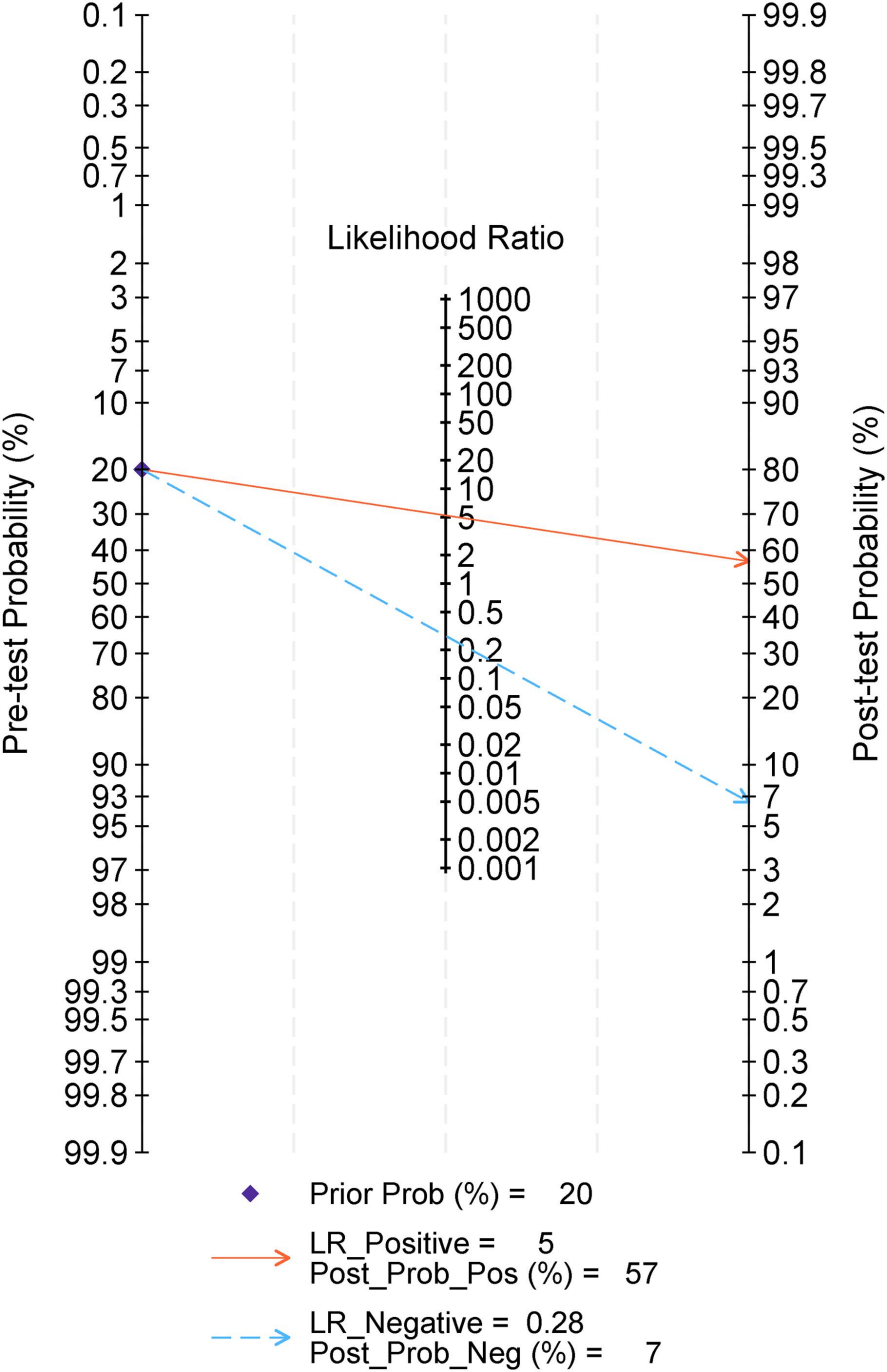
Fagan nomogram for assessing the clinical value of D-dimer.

Fagan's nomogram analysis demonstrated that assuming a pre-test probability of 20%, if the D-dimer test result is positive, the post-test probability of DVT rises to approximately 57%; if the D-dimer test result is negative, the post-test probability of DVT decreases to approximately 7% (Figure 6). This indicates that a negative result holds good clinical value for ruling out DVT, while the diagnostic capability of a positive result is relatively limited.

### Publication bias

**Figure 7:**
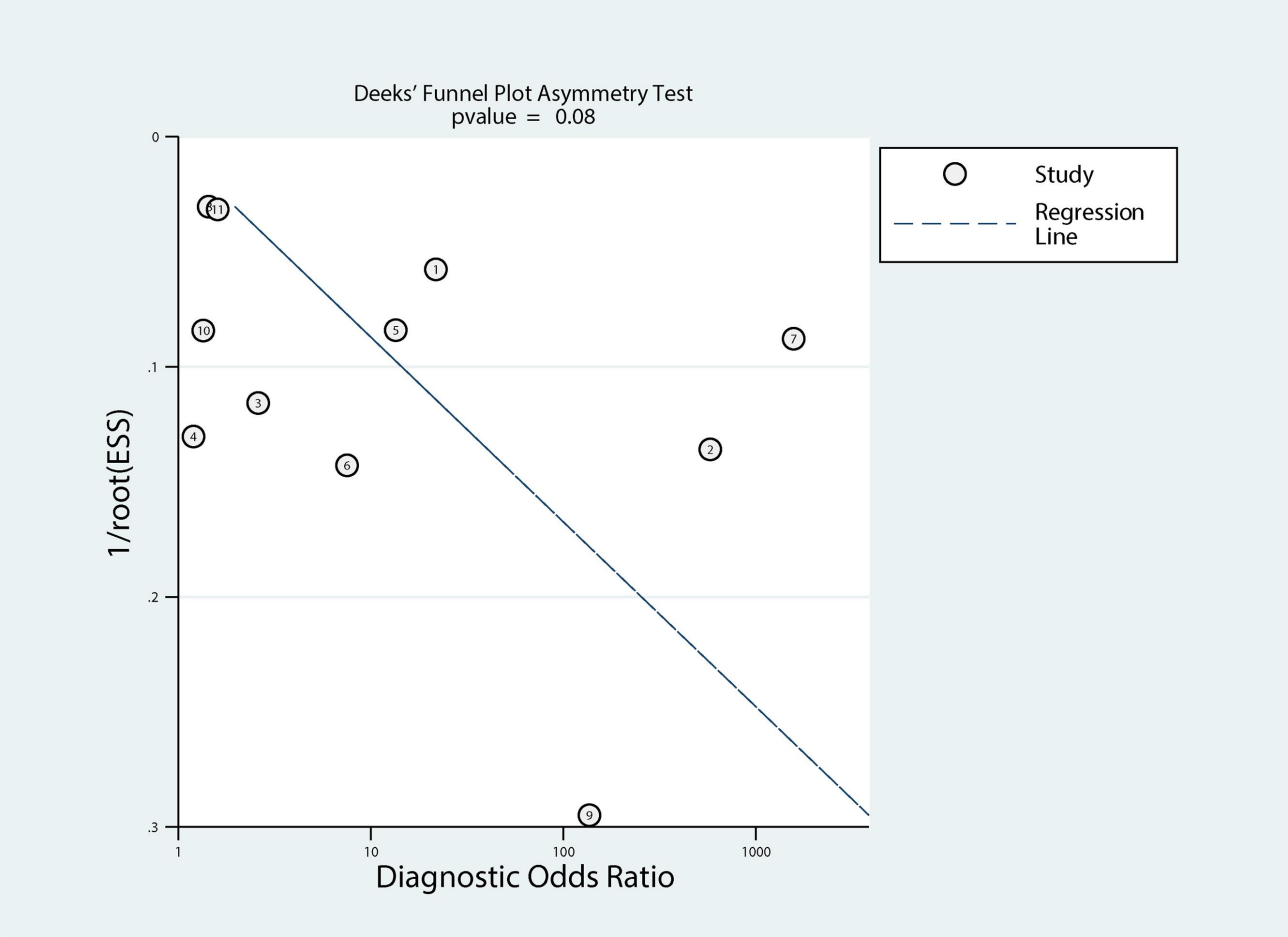
Deeks’ funnel plot for assessing publication bias.

Publication bias was evaluated using Deeks’ funnel plot asymmetry test, and the result showed *P* = 0.08 (Figure 7). Since the *P*-value was less than 0.10, it suggests that there may be some degree of publication bias among the included studies, which should be taken into consideration when interpreting the results.

## Discussion

This study systematically evaluated the diagnostic value of plasma D-dimer for screening deep vein thrombosis (DVT) after joint arthroplasty through a pooled analysis of 11 studies (4, 9–12, 15–20) involving a total of 7,123 patients. The results showed an AUC of 0.83 and a DOR of 18.59 for D-dimer, indicating that an abnormally elevated postoperative D-dimer level has a statistically significant association with DVT formation and possesses potential as an auxiliary diagnostic tool. However, despite overall acceptable parameter performance, the specific performance of the pooled sensitivity (0.76) and specificity (0.86) reveals the complex dilemmas in practical clinical application.

The fundamental limitation of D-dimer in this clinical context stems from two interrelated factors: the underlying pathophysiology of the postoperative state and the consequent methodological challenges in defining an appropriate diagnostic threshold. From a pathophysiological perspective, total joint arthroplasty represents a profound insult to the musculoskeletal and vascular systems. Surgical trauma, bone reaming, cement implantation, and tourniquet application collectively trigger a robust activation of the coagulation cascade and the fibrinolytic system. This results in a predictable, yet highly variable, surge in circulating D-dimer that occurs independently of thrombus formation—a phenomenon best described as ‘non-thrombotic physiological elevation’ (22). Consequently, in the early postoperative period, the majority of patients exhibit D-dimer levels that substantially exceed the conventional venous thromboembolism exclusion threshold (e.g., 0.5 mg/L), regardless of whether DVT is actually present. This creates a situation where the test's specificity is inherently compromised at baseline.

From a methodological standpoint, the absence of a standardized, procedure-specific cut-off value exacerbates this problem. As demonstrated by the wide range of thresholds reported in our included studies (0.185–0.845 mg/L), clinicians and researchers face an unavoidable trade-off. Adherence to a low, conventional cut-off preserves sensitivity but generates an unacceptably high false-positive rate, leading to excessive and costly confirmatory imaging. Conversely, adopting a higher, empirically derived cut-off improves specificity and reduces unnecessary testing, but does so at the direct expense of sensitivity, thereby increasing the risk of missed thrombi—particularly small, distal, or non-occlusive clots. This intrinsic trade-off, dictated by the overlap in D-dimer distributions between DVT-positive and DVT-negative postoperative patients, ultimately precludes the use of a single D-dimer measurement as a standalone diagnostic tool. It is for these reasons that D-dimer in this setting is best conceptualized not as a diagnostic test, but as a component of a broader risk-stratification algorithm.

This study found that the pooled sensitivity of D-dimer was only 0.76, a value lower than the threshold typically required (usually >90%) for a first-line “rule-out” marker in clinical practice. This suggests that relying solely on D-dimer for screening may carry a missed diagnosis risk of approximately 24% (18–20). The reasons for the insufficient sensitivity are multifaceted. On one hand, some included studies used higher cutoff values [e.g., 0.845 mg/L used by Wu, J. et al. (19)] to improve specificity. Such an upward adjustment of the threshold inevitably leads to missed diagnoses of low-burden or muscular venous thrombi. On the other hand, DVT formation is a dynamic process, and a single measurement at a fixed cutoff point may fail to capture early signals of thrombus formation, leading to false negatives (21). Although the pooled specificity reached 0.86, its confidence interval was extremely wide (0.55–0.97), and the between-study heterogeneity was extremely high. This extreme instability profoundly reflects the pathophysiological paradox of D-dimer application following major orthopedic surgery. Surgical trauma, tourniquet use, and bone marrow reaming inevitably trigger a robust systemic inflammatory response and physiological activation of the coagulation-fibrinolytic system (22). This “non-thrombotic physiological surge” creates substantial background noise, causing an overlap in D-dimer levels between DVT and non-DVT patients. Among the included large-sample studies, specificity varied dramatically [e.g., only 0.22 in Zhang, K (20). vs. 1.00 in Xin, H (16).]. This suggests that in clinical application, without correction for factors such as age or surgical type, the false positive rate at the conventional cutoff (0.5 mg/L) may be extremely high, leading to a large number of unnecessary ultrasound examinations.

Further analysis of heterogeneity sources revealed that the choice of cutoff value is a key variable affecting diagnostic performance. The cutoff values in the included studies ranged widely from 0.185 mg/L to 0.845 mg/L (4, 9–12, 15–20). This wide variation provides a classic illustration of the ‘threshold effect’ inherent in diagnostic test accuracy meta-analyses. As the diagnostic threshold is lowered, sensitivity typically increases at the expense of specificity, and vice versa, producing a negative correlation between the two metrics across studies. This phenomenon directly accounts for a substantial proportion of the observed between-study heterogeneity. Lower cutoffs, while preserving sensitivity, often result in unacceptable specificity; studies that introduced age-adjusted formulas or higher cutoff values achieved a better balance between the two. Moreover, the striking disparity in reported cut-off values—spanning nearly a fivefold range—underscores the absence of a consensus regarding the optimal D-dimer threshold in the postoperative setting. This lack of standardization is not merely a statistical nuisance but a genuine clinical challenge; it reflects the fundamental pathophysiological reality that surgical trauma induces a variable and sustained elevation in D-dimer that renders conventional venous thromboembolism exclusion thresholds inapplicable. Consequently, this finding reinforces our conclusion that a rigid ‘one-size-fits-all’ diagnostic cut-off is invalid in this population and that future efforts should prioritize the development of stratified or individualized threshold strategies.

Additionally, differences in surgical type cannot be ignored. Due to tourniquet use, the postoperative D-dimer peak is typically higher following total knee arthroplasty (TKA) than following total hip arthroplasty (THA). Pooled analysis may obscure the optimal diagnostic threshold for different surgical subgroups, indicating that future clinical practice should not adhere to a “one-size-fits-all” standard but rather establish a stratified cutoff system based on surgical site and patient characteristics.

Regarding clinical utility, Fagan nomogram analysis showed that under the assumption of a 20% pre-test probability, a negative D-dimer result (NLR=0.28) could reduce the post-test probability of DVT to approximately 7%. This absolute risk reduction of 13 percentage points represents a clinically meaningful decrease. While a 7% residual probability does not permit definitive exclusion of DVT, it falls within a range where clinicians, in conjunction with a comprehensive clinical assessment and risk stratification (e.g., Caprini score), may reasonably consider deferring immediate ultrasound imaging or withholding empirical anticoagulation in low- to moderate-risk patients. In other words, a negative D-dimer result helps identify a subset of postoperative patients with a sufficiently low post-test likelihood of DVT to safely avoid further resource-intensive diagnostic workup. Conversely, a positive result (PLR=5.24) only increased the post-test probability to 57%, reaffirming that an elevated D-dimer alone lacks diagnostic specificity in this setting and necessitates confirmatory imaging. Thus, the primary clinical value of postoperative D-dimer testing lies not in its diagnostic confirmation but in its capacity, when negative, to inform risk-appropriate decision-making as part of a multimodal screening strategy. Several important stratification factors that may influence the diagnostic performance of D-dimer warrant further discussion, although the current meta-analysis was unable to formally evaluate them due to insufficient reporting in the primary studies. First, gender-specific differences in D-dimer physiology have been documented, with female patients exhibiting significantly higher baseline and postoperative D-dimer levels compared to males, independent of thrombotic events. Consequently, applying uniform cut-off values may systematically underestimate specificity in women while overestimating it in men, underscoring the potential utility of sex-adjusted thresholds. Second, the extent of surgical trauma differs substantially between unilateral and bilateral total knee arthroplasty. Bilateral procedures induce a greater systemic inflammatory and procoagulant response, leading to a more pronounced non-thrombotic D-dimer elevation and, accordingly, a higher false-positive rate. This suggests that distinct diagnostic cut-offs may be required for unilateral vs. bilateral surgeries to optimize test performance. Third, and perhaps most critically, the anatomic location of the thrombus profoundly affects D-dimer sensitivity. The test demonstrates consistently lower sensitivity for distal (calf) deep vein thrombosis compared to proximal DVT, as the fibrin burden in isolated distal clots is often insufficient to generate a D-dimer level exceeding standard or even adjusted cut-off values. This limitation is particularly relevant in the orthopedic setting, where distal DVT constitutes a substantial proportion of postoperative thrombi. Future well-designed prospective studies should prioritize reporting stratified data by these variables to enable more precise, patient-tailored diagnostic algorithms.

In addition to the pathophysiological and methodological limitations discussed above, the diagnostic utility of D-dimer in this population is further confounded by the effects of commonly administered perioperative medications. First, anticoagulants used for thromboprophylaxis, such as rivaroxaban, directly inhibit the coagulation cascade and reduce thrombus formation and propagation. Consequently, thrombi that do develop in anticoagulated patients tend to be smaller in volume and burden, generating lower levels of fibrin degradation products. This pharmacologic attenuation of the very signal that D-dimer is intended to detect substantially impairs its diagnostic sensitivity. Indeed, in a cohort of TKA patients receiving rivaroxaban thromboprophylaxis, the area under the ROC curve for postoperative D-dimer was only 63.5%, with a sensitivity of merely 58.6% at the optimal cutoff value (23). These findings underscore that D-dimer testing is of limited value in patients receiving effective pharmacologic thromboprophylaxis. Second, recombinant human erythropoietin (EPO), frequently administered in the perioperative period to manage anemia and reduce allogeneic blood transfusion requirements, may also influence D-dimer levels. EPO therapy has been shown to increase whole blood viscosity (24) and enhance platelet reactivity and endothelial activation (25), thereby promoting a procoagulant milieu that can elevate D-dimer independent of overt thrombus formation. Given the widespread use of both anticoagulants and erythropoiesis-stimulating agents in contemporary joint arthroplasty practice, the interpretation of postoperative D-dimer results must be undertaken with full awareness of these pharmacologic confounders (26).

Beyond the aforementioned limitations, several methodological concerns further constrain the reliability of our pooled estimates. First, as shown in Table 2, the majority of included studies had a high risk of bias in the patient selection domain. This was largely attributable to their retrospective design, where patient enrollment was often non-consecutive or based on case-control sampling. Such designs are known to overestimate diagnostic accuracy compared to prospective, consecutive cohort studies. Second, although all studies used venography or Doppler ultrasound as the reference standard, some relied on venography—the true gold standard—while others used ultrasound, which has imperfect sensitivity for distal DVT. This variation may have contributed to the observed heterogeneity. Third, the extremely high statistical heterogeneity (I^2^ > 98% for both sensitivity and specificity) indicates that the pooled estimates should be interpreted with caution. While such high heterogeneity is common in diagnostic meta-analyses due to threshold effects, the combination of clinical diversity (different surgical types, timing of measurement, and cutoff values) and methodological limitations (retrospective designs, selection bias) makes it unlikely that a single summary estimate accurately reflects D-dimer's performance across all clinical settings. Therefore, our pooled results should be viewed as providing a general indication of diagnostic potential rather than precise, universally applicable estimates. Clinicians and researchers are encouraged to consider the specific context of their own patient populations and institutional practices when interpreting these findings.

In summary, the current meta-analysis suggests that D-dimer has moderate diagnostic potential for screening DVT after total joint arthroplasty, with a negative result providing clinically useful ruling-out value. However, the pooled estimates are subject to substantial heterogeneity and a high risk of bias in the included studies. These findings should not be interpreted as supporting a universal diagnostic threshold or the use of D-dimer as a standalone test. Instead, D-dimer may be most appropriately employed as one component of a risk-stratified screening strategy, ideally combined with clinical risk assessment tools (e.g., the Caprini score) or stratified, procedure-specific cutoff values. Given the current limitations in the evidence base, the decision to use D-dimer in postoperative DVT screening should be made with full awareness of its poor specificity in this setting and the potential for false-positive results. Future well-designed prospective studies are needed to validate integrated multimodal algorithms and to define optimal, context-specific thresholds before routine clinical adoption.

## Data Availability

The original contributions presented in the study are included in the article/Supplementary Material, further inquiries can be directed to the corresponding author.
