# Supplementary material for: Diagnostic value of D-dimer in screening for deep vein thrombosis after total joint arthroplasty: a meta-analysis
Source: Front Surg. 2026 Jul 14;13:1783183. doi: 10.3389/fsurg.2026.1783183 (PMC13407221; doi:10.3389/fsurg.2026.1783183)
Supplement: Supplementary file 1 [file Supplementaryfile1.docx]

**Supplementary Material 1: Detailed Search Strategies**

| **Database** | **Search Strategy** |
| --- | --- |
| **PubMed** | "D-dimer" OR "D-dimer test" OR "plasma D-dimer" AND "deep vein thrombosis" OR "DVT" AND "total hip arthroplasty" OR "THA" OR "total knee arthroplasty" OR "TKA" OR "femoral head replacement" OR "FHR" AND "diagnostic accuracy" OR "sensitivity" OR "specificity" OR "cut-off value" AND "prospective study" OR "retrospective study" |
| **知网（CNKI** | "D-二聚体" OR "D-二聚体检测" OR "血浆D-二聚体" AND "深静脉血栓" OR "DVT" AND "全髋关节置换术" OR "THA" OR "全膝关节置换术" OR "TKA" OR "股骨头置换术" OR "FHR" AND "诊断准确性" OR "敏感性" OR "特异度" OR "截断值" AND "前瞻性研究" OR "回顾性研究" |
| **Willey library** | 'D-dimer' OR 'D-dimer test' OR 'plasma D-dimer' AND 'deep vein thrombosis' OR 'DVT' AND 'total hip arthroplasty' OR 'THA' OR 'total knee arthroplasty' OR 'TKA' OR 'femoral head replacement' OR 'FHR' AND 'diagnostic accuracy' OR 'sensitivity' OR 'specificity' OR 'cut-off value' AND 'prospective study' OR 'retrospective study' |
| **Web of Science** | TS=("D-dimer" OR "D-dimer test" OR "plasma D-dimer") AND TS=("deep vein thrombosis" OR "DVT") AND TS=("total hip arthroplasty" OR "THA" OR "total knee arthroplasty" OR "TKA" OR "femoral head replacement" OR "FHR") AND TS=("diagnostic accuracy" OR "sensitivity" OR "specificity" OR "cut-off value") AND TS=("prospective study" OR "retrospective study") |
| **万方（Wanfang）** | "D-二聚体" OR "D-二聚体检测" OR "血浆D-二聚体" AND "深静脉血栓" OR "DVT" AND "全髋关节置换术" OR "THA" OR "全膝关节置换术" OR "TKA" OR "股骨头置换术" OR "FHR" AND "诊断准确性" OR "敏感性" OR "特异度" OR "截断值" AND "前瞻性研究" OR "回顾性研究" |
| **维普（VIP）** | "D-二聚体" OR "D-二聚体检测" OR "血浆D-二聚体" AND "深静脉血栓" OR "DVT" AND "全髋关节置换术" OR "THA" OR "全膝关节置换术" OR "TKA" OR "股骨头置换术" OR "FHR" AND "诊断准确性" OR "敏感性" OR "特异度" OR "截断值" AND "前瞻性研究" OR "回顾性研究" |
